# Bromelain Decreases Neutrophil Interactions with P-Selectin, but Not E-Selectin, In Vitro by Proteolytic Cleavage of P-Selectin Glycoprotein Ligand-1

**DOI:** 10.1371/journal.pone.0078988

**Published:** 2013-11-11

**Authors:** Jessica M. Banks, Christine T. Herman, Ryan C. Bailey

**Affiliations:** Department of Chemistry, University of Illinois at Urbana-Champaign, Urbana, Illinois, United States of America; University of Bern, Switzerland

## Abstract

Stem bromelain, a cysteine protease isolated from pineapples, is a natural anti-inflammatory treatment, yet its mechanism of action remains unclear. Curious as to whether bromelain might affect selectin-mediated leukocyte rolling, we studied the ability of bromelain-treated human neutrophils to tether to substrates presenting immobilized P-selectin or E-selectin under shear stress. Bromelain treatment attenuated P-selectin-mediated tethering but had no effect on neutrophil recruitment on E-selectin substrates. Flow cytometric analysis of human neutrophils, using two antibodies against distinct epitopes within the P-selectin glycoprotein ligand-1 (PSGL-1) active site, revealed that bromelain cleaves PSGL-1 to remove one of two sites required for P-selectin binding, while leaving the region required for E-selectin binding intact. These findings suggest one molecular mechanism by which bromelain may exert its anti-inflammatory effects is via selective cleavage of PSGL-1 to reduce P-selectin-mediated neutrophil recruitment.

## Introduction

Inflammation is a complex physiological process involving numerous receptor-ligand interactions between leukocytes and the endothelial lining of the blood vessel that ultimately lead to the trafficking of leukocyte subsets throughout the body [1]. Numerous diseases are associated with dysregulated inflammation, including rheumatoid arthritis, asthma, psoriasis, thrombotic disorders, cancer, and autoimmune disease [2].

Bromelain is a mixture of several cysteine proteases isolated from pineapple extracts, and is taken as a complementary anti-inflammatory treatment [3]. Bromelain is known to alter multiple cell surface molecules involved in the adhesion and activation of leukocytes [4] leading to anti-inflammatory, fibrinolytic, and anti-thrombotic effects *in vivo* and *in vitro* [5]–[7]. Previous reports have investigated the effects of bromelain treatment on neutrophil migration in response to chemokines [4], [8]. However, bromelain’s ability to alter cell surface molecules involved in the initial tethering and rolling of leukocytes on the inflamed endothelium has not been investigated to date. Among the enzymes present in bromelain extract is stem bromelain, which was used in this study and will be simply referred to as bromelain herein.

**Figure 1:**
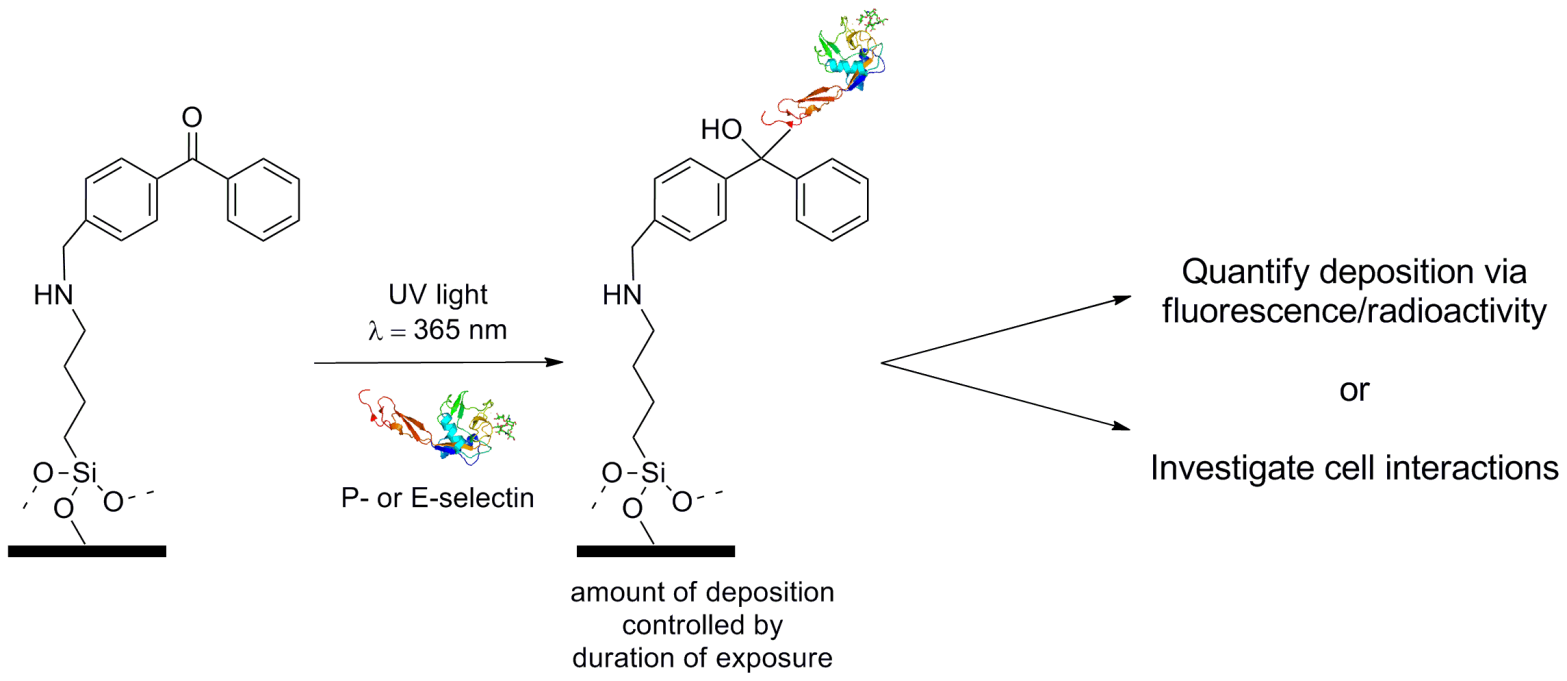
Substrates for cell interactions studies were fabricated via direct, photochemical functionalization. Glass slides chemically-modified to present benzophenone moieties were immersed in a solution containing the protein of interest–here P- or E-selectin. Photoimmobilization is initiated by exposure to 365 nm light, which results to covalently bound protein through a radical generation mechanism. Importantly, the density of immobilized protein can be systematically defined by carefully controlling the exposure time. Substrates, prepared in batches, were then subsequently used to quantify deposition density (via fluorescence or radioimmunoassay) or utilized to study the effects of bromelain treatment on primary human neutrophil-selectin interactions.

Endothelial-expressed P-selectin and E-selectin play central roles during the initiation of an inflammatory response. When the endothelium receives distress signals from underlying tissue, P-selectin is the first biomolecule deployed from intracellular storage pools to the luminal surface [9], [10]. The interaction between P-selectin and its primary leukocyte-expressed ligand, P-selectin glycoprotein ligand-1 (PSGL-1), supports neutrophil rolling along the surface of the blood vessel [11]. E-selectin, which binds to PSGL-1 among other ligands [12] is also presented on the endothelium during the inflammatory response, but its expression is largely controlled by translation [13] and its presentation temporally lags behind that of P-selectin *in vivo* [9]. Given these temporal selectin expression dynamics, we felt it would be beneficial to independently probe bromelain’s effects on neutrophil interactions with substrates presenting each of these glycoproteins. To achieve this, we utilized a photochemical surface modification strategy [14] developed in our lab to generate substrates presenting controlled densities of P-selectin or E-selectin [15], and then used these substrates to investigate the effect of bromelain treatment on the ability of human neutrophils to tether and roll in flow assays (Figure 1).

Neutrophils are among the first responders that rapidly accumulate at sites of inflammation and thus a potential key player in the initial steps of the immune response [16]. Flow cytometric expression analysis of two ligands involved in leukocyte recruitment mediated by P-selectin and E-selectin, PSGL-1 and cutaneous lymphocyte antigen (CLA), respectively, was performed to determine the effects of bromelain treatment on ligand expression. The results reveal a site-specific proteolysis through which bromelain treatment abolishes interactions between neutrophils and immobilized P-selectin, but not E-selectin, under conditions of physiological shear stress *in vitro*, suggesting another molecular mechanism through which bromelain may act as an anti-inflammatory agent.

## Results and Discussion

Interestingly, we found that bromelain treatment of neutrophils nearly eliminated their ability to interact with P-selectin presented on substrates *in vitro*, while E-selectin-mediated interactions are unaffected. The observations from neutrophil flow assays were complemented by dose-dependent effects of bromelain treatment on the expression of P-selectin and E-selectin ligands using flow cytometry. Our findings suggest that the anti-inflammatory effects of bromelain may be attributed in part to its ability to proteolytically process PSGL-1 and thereby reduce the number of cells interacting with P-selectin presented on the inflamed endothelium during the initial phases of an inflammatory response.

**Figure 2:**
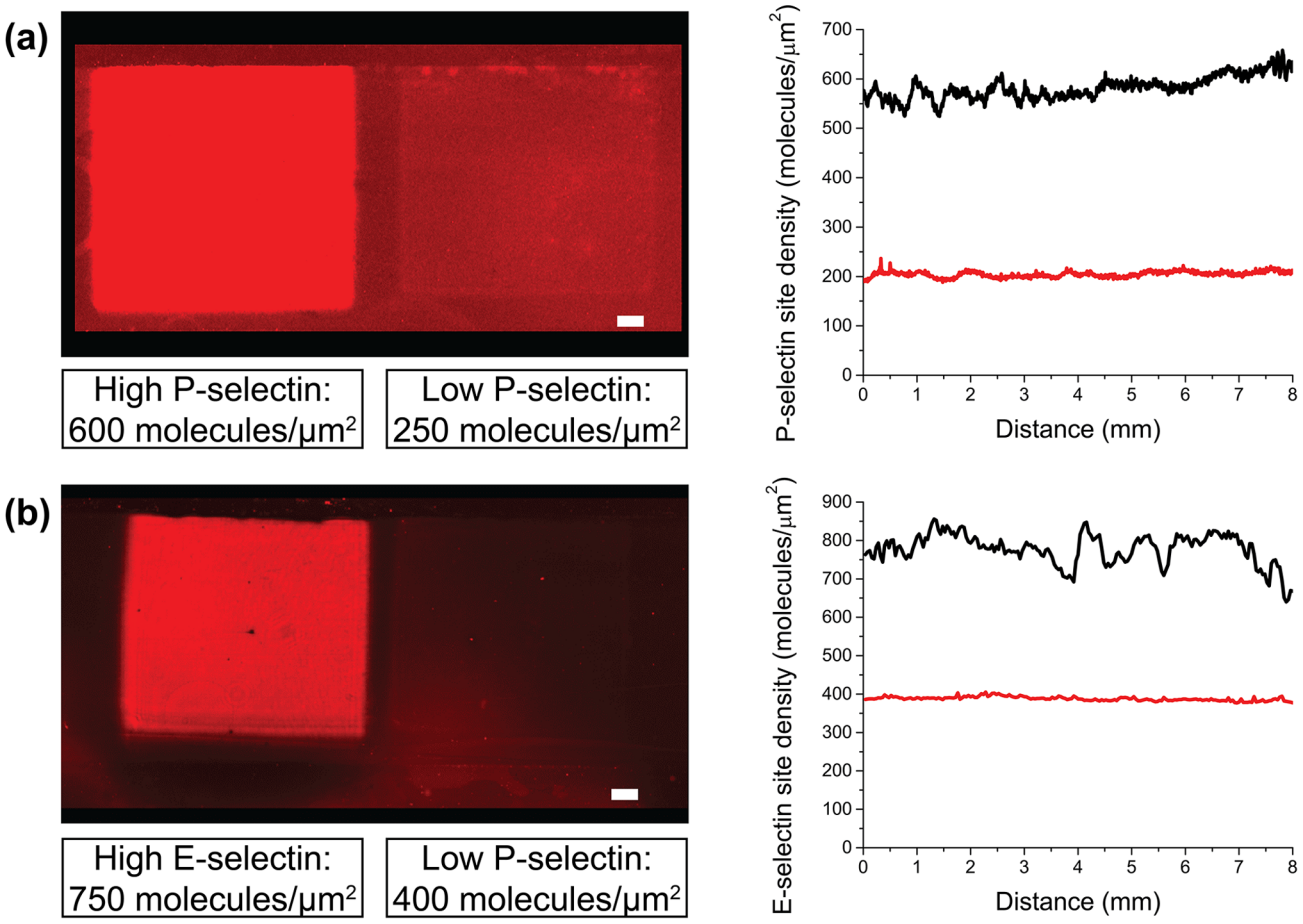
Fluorescence images and plots of average site density from substrates presenting photoimmobilized selectins. (a) Sites densities for P-selectin substrates were determined using a previously reported radioimmunoassay [15]. (b) A similar radioimmunoassay for E-selectin (Figure S1 in File S1) was used to determine site densities of E-selectin substrates. Scale bar = 1 mm.

Using a molecularly general method previously developed in our lab for the covalent and controllable photochemical immobilization of biomolecules on planar glass substrates [14], we generated and characterized substrates presenting defined “high” and “low” levels of P- or E-selectin (Figures 1–2 and Figure S1 in File S1). Substrates were characterized with fluorescence imaging and average fluorescence intensity values were converted into biomolecule site densities using an established fluorescence-radioactivity correlation for P-selectin [15] and E-selectin (Figure S2 in File S1).

**Figure 3:**
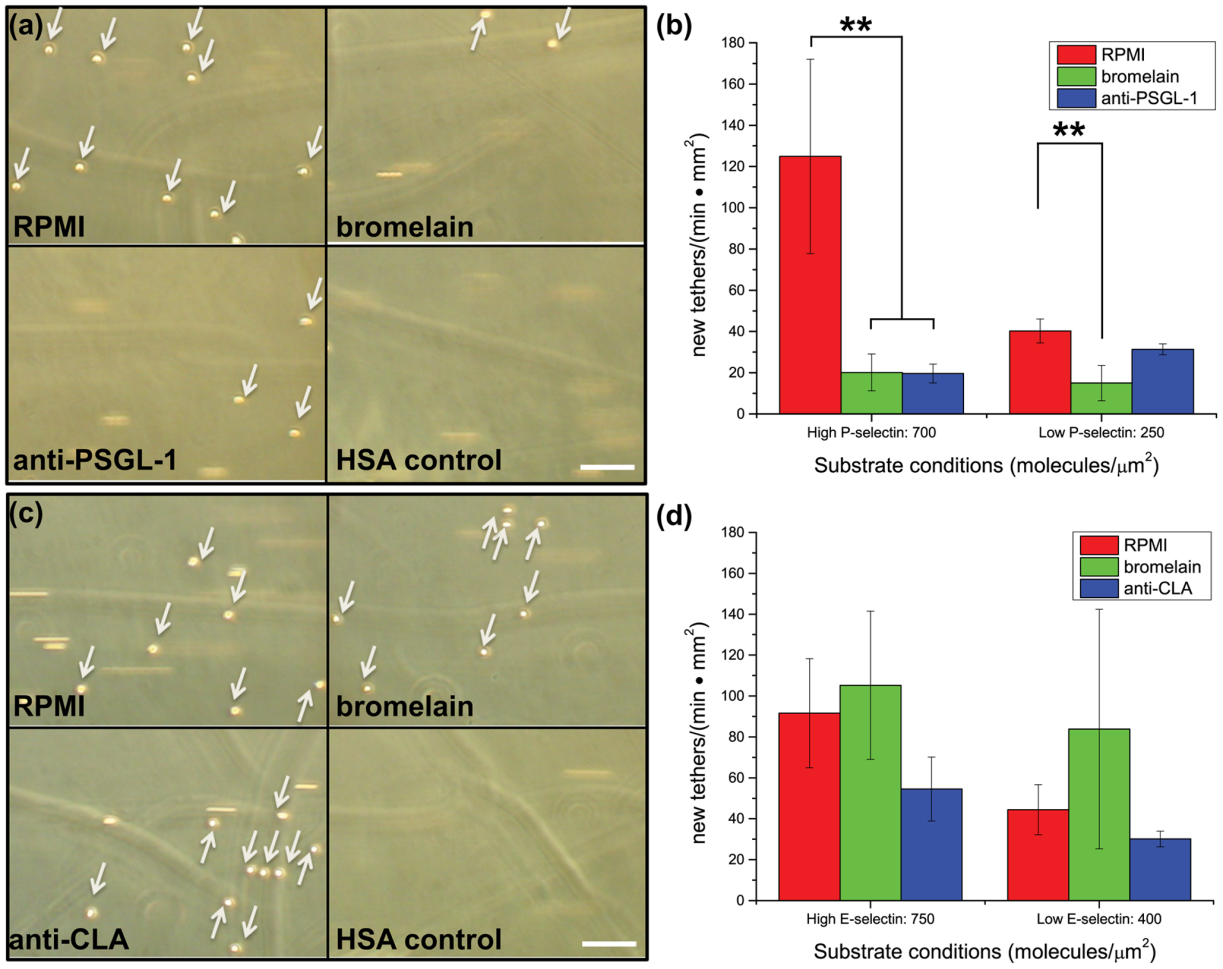
Data from flow assays with neutrophils treated with RPMI or bromelain. Bromelain attenuates neutrophil tethering on immobilized P-selectin but has no effect on E-selectin-mediated tethering. (a) Snapshots from representative flow assay videos show neutrophils interacting with substrates presenting immobilized P-selectin. Arrows point to cells interacting with the substrate under conditions of shear stress. (b) Data from primary human neutrophil flow assays with P-selectin substrates reveal that the number of interacting cells is decreased when neutrophils are treated with 50 µg/mL bromelain in RPMI on regions of “high” and “low” immobilized P-selectin. (** *p*<0.05) Treatment with saturating levels of anti-PSGL-1 clone KPL-1 significantly decreased P-selectin mediated tethering on high P-selectin substrates (** *p*<0.05). (c) Snapshots from representative flow assay videos show neutrophils interacting with substrates presenting immobilized E-selectin with arrows indicating interacting cells. (d) Data from primary human neutrophil flow assays with E-selectin substrates reveal that the number of interacting cells is not significantly affected when neutrophils are treated with 50 µg/mL bromelain in RPMI on regions of “high” or “low” immobilized E-selectin. Treatment with saturating levels of anti-CLA clone HECA-452 had no significant effect on neutrophil tethering to immobilized E-selectin. Neutrophils failed to interact with HSA-blocked control substrates. Data represent the average of *n* = 3 donors (±SEM). Scale bar: 80 µm.

Figure 3 shows neutrophil flow assay results using primary human neutrophils. For the first set of flow assays, human neutrophils were isolated (>90% pure, >95% viable) and flowed at a constant shear stress over substrates presenting immobilized P-selectin at high and low site densities. The number of interacting cells was significantly reduced when neutrophils were treated with 50 µg/mL bromelain, compared to a control sample (*p*<0.05). Incubation of control cells with saturating levels of a monoclonal antibody (mAb) against PSGL-1, clone KPL-1, which recognizes an epitope encompassing three sulfated tyrosines in the active site of PSGL-1, similarly resulted in a decrease in the number of interacting cells, suggesting that the decreased interactions are due to reduced PSGL-1 association with underlying P-selectin. In the case of bromelain treatment, this is caused by proteolytic cleavage of PSGL-1 so as to remove the region of the molecule required to engage P-selectin.

**Figure 4:**
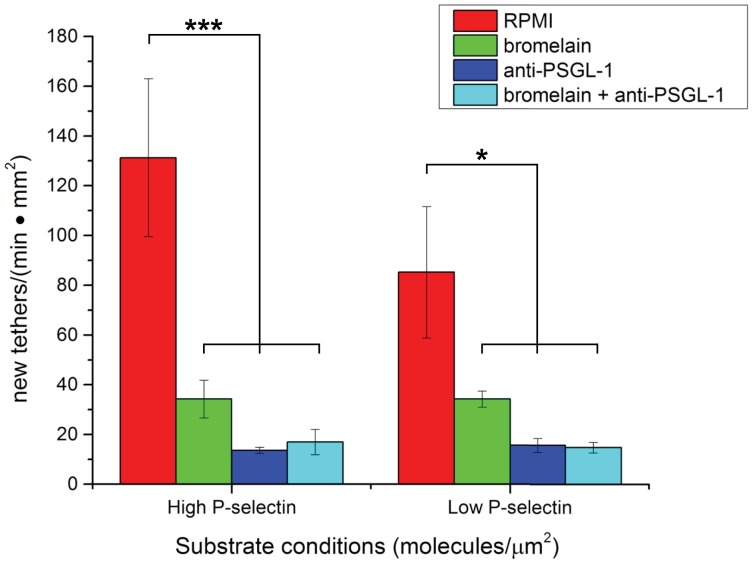
Blocking of PSGL-1 with a monoclonal anti-PSGL-1 antibody prevents subsequent proteolytic cleavage by bromelain thereby preventing further reduction in tethering on P-selectin. Data from flow assays with differentiated HL-60 promyelocytes treated with bromelain, blocking antibody, both, or untreated. For both “high” (*** *p*<0.001) and “low” (* *p*<0.10) P-selectin substrates, bromelain, blocking antibody, and combination treatments reduced substrate interactions. In both cases, combination treatment did not significantly further reduce interactions beyond that of bromelain treatment alone. Data represent the average of *n* = 3 independent P-selectin substrates (±SEM).

It is well established that PSGL-1 is the predominant and most well-characterized binding partner of P-selectin. [17] However, we wanted to validate the specific targeting of PSGL-1 by bromelain as an effector of rolling and tethering on P-selectin substrates and therefore performed an additional control experiment. This experiment utilized neutrophil-like HL-60 promyelocytes, which also tether and roll on P-selectin surfaces via PSGL-1, and a monoclonal anti-PSGL-1 blocking antibody (clone KPL-1) with and without bromelain treatment. As shown in Figure 4, treatment with the blocking antibody alone led to a marked decrease in the number of tethering events on both “high” and “low” P-selectin substrates. Importantly, HL-60 cells that were treated with bromelain after being blocked with the antibody did not show a further reduction in substrate interaction, confirming the specific action of bromelain on PSGL-1 as it leads to reduced interactions with P-selectin surfaces.

P-selectin is the primary molecular player that controls initial neutrophil interactions with the inflamed endothelium; however E-selectin is also known to play a key role in mediating leukocyte rolling. PSGL-1 is a ligand for both P- and E-selectin and therefore we wanted to see whether bromelain treatment similarly abolished neutrophil interactions on E-selectin. Isolated human neutrophils were flowed over substrates presenting two different site densities of immobilized E-selectin. In contrast to experiments on P-selectin-presenting substrates, bromelain treatment had no significant effect on neutrophil tethering to immobilized E-selectin. The cutaneous lymphocyte antigen (CLA) glycan moiety pendant on PSGL-1 [18], [19] and other ligands [20], plays a role in leukocyte tethering and rolling mediated by E-selectin [19]. To see if neutrophil interactions with immobilized E-selectin could be altered by blocking CLA on all E-selectin ligands, we incubated neutrophils with saturating levels of HECA-452, a widely used mAb that targets CLA. Not surprisingly, this blocking antibody reduced tethering to immobilized E-selectin (Figure 3c–d), though the effect was not as significant as that for the anti-PSGL-1 blocking on P-selectin substrates. The incomplete inhibition of tethering on E-selectin after anti-CLA blocking reflects the fact that E-selectin, unlike P-selectin, has a diverse range of potential binding partners and thus simply blocking one binding epitope does not abolish all interactions, a finding consistent with the literature [21]. Overall, neutrophil flow assays with E-selectin-presenting substrates reveal that bromelain does not proteolytically abolish all interactions between neutrophils and E-selectin. Importantly, the sulfated tyrosine residue epitope targeted by KPL-1 is not required for recognition of PSGL-1 by E-selectin [22] and so the unaffected interactions with this substrate after bromelain treatment is consistent with a molecularly specific cleavage of PSGL-1, but also supports E-selectin recognition by ligands besides PSGL-1.

Neutrophils flowed across simple human serum albumin-blocked substrates, without P- or E-selectin, showed no interactions which established that observed cell-substrate interactions were the result of specific receptor-ligand interactions.

To further investigate the molecular mechanism by which bromelain treatment regulates the initial phases of neutrophil tethering, we performed flow cytometric analyses to determine how expression of PSGL-1, which binds to both P-selectin and E-selectin, is modulated by bromelain treatment. There are two distinct structural domains of PSGL-1 that are required for interactions with each of the selectins and our flow cytometric analysis utilized two mAbs specific for these regions. Antibody KPL-1 recognizes a region that contains the sulfated tyrosine motif required for interactions with P-selectin, but not E-selectin [22]. This specificity was previously demonstrated as incubation of leukocytes with the KPL-1 antibody completely blocked interactions with P-selectin without affecting leukocyte recognition of E-selectin [23]. Antibody CHO131 recognizes a sialyl-Lewis^x^–bearing a core 2 O-glycan that is required for interaction of neutrophils with both P-selectin and E-selectin [24].

**Figure 5:**
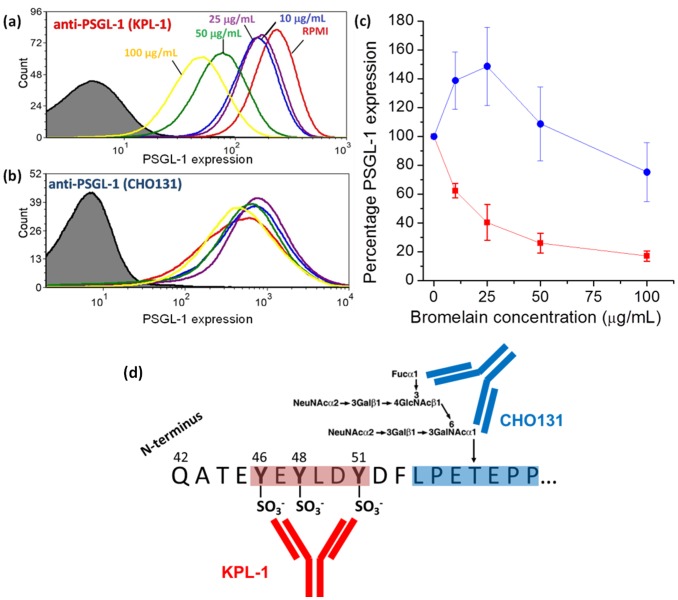
Flow cytometric analysis reveals bromelain cleaves PSGL-1 within its active site in a dose-dependent manner. To analyze neutrophil expression of PSGL-1, the primary ligand for P-selectin, we used two anti-PSGL-1 antibodies that recognize distinct structural motifs within the PSGL-1 active site. (a) Representative data from an analysis with clone KPL-1 reveals an 80% decrease in PSGL-1 expression following treatment with 100 µg/mL bromelain. (b) Representative data from an analysis with clone CHO131 reveals a slight increase followed by a decrease in PSGL-1 expression back to initial levels as bromelain concentrations increase from 0 to 100 µg/mL. (c) The average PSGL-1 expression levels of neutrophils from *n* = 3–4 donors (±SEM) plotted as a function of bromelain concentration, suggesting that bromelain cleaves PSGL-1 at a position between the two epitopes recognized by the site-specific antibodies.

Flow cytometric analysis using these antibodies suggests that bromelain is able to specifically cleave PSGL-1 at a site in between the epitopes for clones KPL-1 and CHO131 (Figure 5). Analysis with clone KPL-1 revealed that the level of the sulfated tyrosine motif is reduced by ∼80% as the dose of bromelain exposure was increased from 0 to 100 µg/mL. However, analysis with clone CHO131 showed that the levels of this epitope were not significantly decreased at all concentrations of bromelain tested. In fact, CHO131 epitope expression moderately increased at low bromelain concentrations before showing a slight decrease at higher concentrations. While we do not completely understand the significance of this increase, it is clear that bromelain is able to directly and dramatically attenuate the sulfated tyrosine motif needed for P-selectin tethering while leaving significant expression of the sLe^x^ glycan needed for E-selectin’s interaction with PSGL-1. Treatment of human neutrophils with deactivated bromelain had no significant effect on neutrophil expression of PSGL-1 (Figure S3 in File S1), suggesting that enzyme activity is required to induce the changes in surface expression of PSGL-1 that we observed following treatment with active bromelain. The molecularly-specific processing of PSGL-1 at a position that down-regulates interactions with P-selectin, yet upstream of the sialyl-Lewis^x^–bearing core 2 O-glycan structure involved in E-selectin interactions, suggests that bromelain may selectively affect the very initial phases of neutrophil recruitment.

**Figure 6:**
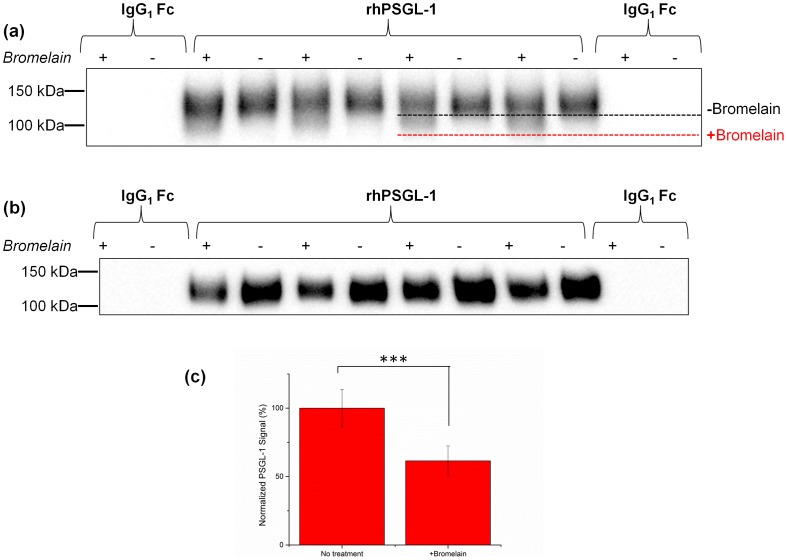
Bromelain proteolytically cleaves PSGL-1. Western blot analysis of untreated PSGL-1 and PSGL-1 treated with bromelain. (a) Western blot probed with a polyclonal antibody recognizing PSGL-1 reveals a decrease in molecular weight after treatment with bromelain. (b) Western blot probed with antibody KPL-1 after treatment with bromelain reveals a 39% decrease in the amount of PSGL-1 corresponding to cleavage of the KPL-1 epitope. (c) Quantification of band intensity shown in figure 6b reveals that bromelain treatment significantly reduces PSGL-1 band intensity (*** *p*<0.001). Data are expressed as the mean ± SD of *n* = 4 replicates. Data are representative of results obtained in three independent experiments.

As additional verification of PSGL-1 processing by bromelain, we performed Western blot analysis of the protein after exposure to the enzyme. Figure 6 compares the 100–150 kDa band of an immunoblot of untreated PSGL-1 and PSGL-1 treated with bromelain. When probed with a polyclonal antibody, no statistically significant difference in intensity was observed but an overall reduction in molecular weight was apparent. When visualized by staining with anti-PSGL-1 clone KPL-1, which recognized the N-terminal portion of the protein that present the sulfated tyrosine residues, there was a significant reduction in the band intensity for bromelain treatment compared to the untreated sample (*p*<0.05). These results are consistent with flow cytometry analysis that showed a marked reduction in the N-terminal component of PSGL-1 using the same antibody clone. As a control, human IgG1 Fc was left untreated or treated identically with bromelain and immunoblotted alongside the PSGL-1 Fc chimera. In both blots, no bands were observed in either IgG1 Fc lanes, confirming that changes in PSGL-1 band intensity are not due to interference from the Fc chimera portion of the recombinant protein.

We also determined the effects of bromelain treatment on expression levels of CLA, a carbohydrate epitope shared by sialyl-Lewis^x^ and sialyl-Lewis^a^ structures that has been found to be present on PSGL-1 and other E-selectin ligands [20]. To characterize CLA expression on neutrophils, we used clone HECA-452, and this analysis revealed that CLA expression declines in a nearly linear fashion by up to 20% as the dose of bromelain increases from 0 to 100 µg/mL (Figure S4 in File S1). Similar to flow cytometry analysis of PSGL-1 expression, treatment of human neutrophils with deactivated bromelain caused no significant changes in the surface levels of CLA (data not shown).

In this study we used a combination of molecular biology and controlled chemical surface modification to reveal new insight into one potential molecular mechanism by which bromelain can regulate immune response. We found that bromelain cleaves PSGL-1, P-selectin’s primary ligand that is involved in the onset of selectin-mediated leukocyte rolling during inflammation, resulting in the attenuation of neutrophil recruitment on immobilized P-selectin, but not E-selectin, *in vitro*. While the levels of bromelain utilized in this study are higher than that known to be achievable in serum, previous studies on the effects of bromelain treatment on leukocyte migration and inflammation established the value of *in vitro* assays using similarly high relative bromelain concentrations. [4], [5] Therefore the basic mechanistic insight gleaned from this study, as well as future efforts to understand neutrophil-selectin interactions both *in vitro* and *in vivo*, may reveal additional key insights into the molecular mechanism underlying bromelain’s anti-inflammatory properties.

## Methods

### Ethics Statement

Blood was obtained from healthy adults who gave informed consent. Written consent was obtained and recorded from all volunteers. This protocol and consent procedure was approved as “*Isolation of human blood cells for rolling and adhesion studies and molecular analysis*” by the Institutional Review Board at the University of Illinois at Urbana-Champaign entitled (IRB #11117).

### Materials

All chemicals were purchased from Sigma-Aldrich, unless otherwise noted.

### Photochemical Immobilization of P-selectin or E-selectin on Glass Substrates

Substrates presenting two regions of distinct site densities of immobilized P-selectin or E-selectin were prepared using a previously reported photopatterning method [14]. Briefly, cleaned glass microscope slides were functionalized with 4-(triethoxysilyl)butyl aldehyde (Gelest), incubated with 20 mM 4-benzoyl benzylamine hydrochloride (Matrix Scientific) and 200 mM NaCNBH_3_, followed by immersion in aldehyde-blocking buffer (200 mM ethanolamine, 0.1 M Tris, pH 7), rinsing water, methanol, and ethanol. P-selectin and E-selectin (R&D Systems) were freshly diluted to 2.5 µg/mL from concentrated stocks into PBS with Ca^2+^ and Mg^2+^ (Sigma). BP-modified substrates were assembled into a rectangular parallel-plate flow chamber (GlycoTech) with a silicone gasket (127 µm thickness). Protein photoimmobilization was enabled through the use of an Ar ion laser (Coherent Innova 90-4, Laser Innovations, 351.1–363.8 nm), whose Gaussian beam profile was converted to a flat-top profile using a π-Shaper (MT-Berlin), and expanded to a 1 cm^2^ area using beam-expanding optics (ThorLabs, 14 mW/cm^2^). At least six replicate substrates were generated presenting areas of high (650–750 molecules/µm^2^) and low (250–400 molecules/µm^2^) protein site density by exposing two distinct regions of each substrate for 3 or 30 seconds (for P-selectin) and for 6 or 60 seconds (for E-selectin). Substrates for flow assays were stored in 0.5% human serum albumin (HSA) in Hank’s Buffered Saline Solution with Ca^2+^ and Mg^2+^ and 10 mM HEPES (pH 7.4, HBSS/HEPES) until use, while the remaining replicate substrates were incubated with fluorescently labeled antibodies and visualized with a fluorescence slide scanner (Figure S1 in File S1, GenePix 4000B, MDS Analytical Technologies), as previously reported for P-selectin [15]. Data for E-selectin quantitation was obtained for this study (Figure S2 in File S1).

### Neutrophil Flow Assays on Substrates Presenting P-selectin and E-selectin

Substrates presenting P-selectin or E-selectin (Figures 2 and S1) were utilized in flow assays with human neutrophils using a previously described methodology [15], shown schematically in Figure 1. Human neutrophils were isolated from whole blood by density centrifugation using Ficoll-Paque, followed by selection with EasySep Human Neutrophil Enrichment Kit (STEMCELL Technologies).

Neutrophil viability was determined with trypan blue staining. Neutrophil purity was determined by flow cytometry using two fluorescently labeled antibodies against neutrophil surface markers CD16 and CD66b (STEMCELL Technologies, Figure S5 in File S1). One sample was resuspended in a solution of 50 µg/mL bromelain in RPMI, and the other two samples were resuspended in RPMI. All samples were incubated for 30 min at 37°C, washed with HSA/HBSS/HEPES, and pelleted for 5 min at 500 rcf. One of the RPMI-treated neutrophil samples was resuspended in 30 µg/mL anti-PSGL-1 (clone KPL-1) or anti-CLA (clone HECA-452) and incubated at room temperature for 30–60 min before being diluted to 0.5×10^6^ cells/mL for flow assay experiments. The remaining cell pellets were immediately resuspended in HBSS/HEPES with 0.5% HSA to 0.5×10^6^ cells/mL. All samples were used in flow assay experiments within 2–3 hours of isolation.

Neutrophils were introduced into the chamber at 3 mL/min (9.3 dyn/cm^2^) for 1 min, then the flow rate was dropped to 400 µL/min (1.28 dyn/cm^2^). After 1 min, 20–30 sec videos were acquired at 4–5 positions in each area of the substrate. Neutrophils from three donors were used to perform independent experiments on three replicate substrates for each protein. For each substrate, the number of new tethers formed per unit area and time was determined by counting the number of cells that interacted with the surface. Cells that were already rolling or adhered upon the surface were not included in the analysis. HSA-blocked BP-modified substrates were used as control substrates to verify that the observed interactions were due to neutrophil interactions with immobilized P-selectin or E-selectin.

### Differentiated HL-60 Flow Assays on Substrates Presenting P-selectin

HL-60 promyelocytes (ATCC) were differentiated for 6 days following addition of 1.3% dimethyl sulfoxide. Cell viability and treatment with RPMI, bromelain, or anti-PSGL-1 (clone KPL-1) was performed as described above for neutrophil flow assays. For double treatment with bromelain and anti-PSGL-1 (clone KPL-1), the sample was resuspended in 30 µg/mL antibody and incubated at room temperature for 10 min before incubation with bromelain for 30 min at 37°C. Flow chamber assays were performed as described for neutrophils on three replicate P-selectin substrates.

### Flow Cytometric Analysis of Bromelain’s Effect on Cell Surface Molecules

Human neutrophils were isolated from whole blood as described above. Neutrophils were prepared for analysis of expression of PSGL-1 and CLA following treatment with 0, 10, 25, 50, or 100 µg/mL bromelain in RPMI cell culture medium for 30 min at 37°C. In some experiments, an additional aliquot of cells were treated with chemically deactivated bromelain, which was prepared by reacting bromelain with dithiothreitol and iodoacetamide, followed by treatment with a proteinase cocktail inhibitor solution containing E-64, according to manufacturer’s protocols. Following incubation with bromelain or RPMI, cells were washed with HSA/HBSS/HEPES and were pelleted for 5 min at 500 rcf. Neutrophils were then blocked for 15 min with 3% HSA in HBSS/HEPES and incubated for 30 min with primary monoclonal antibodies and fluorescently labeled secondary antibodies at final concentrations of 20 and 10 µg/mL, respectively. For detection of cell-surface PSGL-1, we used mouse anti-human PSGL-1 clone KPL-1 (Millipore), and mouse anti-human PSGL-1 clone CHO131 (R&D Systems). For detection of CLA, clone HECA-452 (BioLegend) was used. For all samples, we used secondary antibody PE-conjugated goat anti-mouse IgG (Invitrogen). Control samples were incubated with the PE-labeled secondary antibody alone. Solutions composed of primary and secondary antibodies were preincubated for at least 1 hr prior to incubation with cells. Cells were analyzed with a BD FACSCanto II cytometer (BD Biosciences). Fluorescence data from neutrophil populations were plotted in histogram form (FCS Express, BD Biosciences), and the average fluorescence intensity from each sample was plotted as the percentage of the antigen expression relative to the RPMI-treated sample as a function of bromelain concentration.

### Western Blot Analysis of Proteolytic Processing of Recombinant PSGL-1 by Bromelain

Equimolar amounts of recombinant human PSGL-1 Fc chimera (R&D Systems) or recombinant human IgG1 Fc (R&D Systems) were treated with bromelain at a final concentration of 10 µg/mL for 30 minutes at 37°C. PSGL-1 was left untreated under the same conditions. Samples were electrophoresed on a 4–20% SDS-polyacrylamide gel (Bio-Rad) and immunoblotted on PVDF membranes (Millipore). The 100–150 kDa band of PSGL-1 (consistent with manufacturer specifications) was visualized with anti-PSGL-1 antibody clone KPL-1, which recognizes the sulfated tyrosine residues near the N-terminus, or a polyclonal PSGL-1 antibody (R&D Systems). After incubation with relevant secondary HRP-linked antibody, the blot was visualized using enhanced chemiluminescent substrate (Pierce). Digital images were taken using an ImageQuant LAS 4010 biomolecular imager (GE) and analyzed using ImageQuant TL software (GE).

### Statistical Analysis

Groups were compared using one-way ANOVA with Tukey’s HSD test for post-hoc comparison of more than two groups. For western blot data in Figure 6c, student’s unpaired *t* test was used to compare results. Data were analyzed with GraphPad Prism software and *p*-values are provided.

## Supporting Information

File S1 (PDF)
