# Supplementary material for: Bromelain Decreases Neutrophil Interactions with P-Selectin, but Not E-Selectin, In Vitro by Proteolytic Cleavage of P-Selectin Glycoprotein Ligand-1
Source: PLoS One. 2013 Nov 11;8(11):e78988. doi: 10.1371/journal.pone.0078988 (PMC3823987; doi:10.1371/journal.pone.0078988)
Supplement: File S1 — (PDF) [file pone.0078988.s001.pdf]

Supplemental Data

Bromelain Decreases Neutrophil Interactions with P-selectin, but not E-Selectin, *In Vitro* by Proteolytic Cleavage of P-selectin Glycoprotein Ligand-1

Jessica M. Banks,[†] Christine T. Herman,[†] and Ryan C. Bailey[*]

From the Department of Chemistry, University of Illinois at Urbana-Champaign, Urbana, IL 61801, USA

Supplemental Data:

Figure S1: Average fluorescence intensity and site density determination for P-selectin and E-selectin substrates.

Figure S2: Fluorescence intensity and site density correlation determined for photoimmobilized E-selectin on BP-modified substrates.

Figure S3: Flow cytometry analysis of CD16 and CD66b—markers for neutrophil purity.

Figure S4: Flow cytometry analysis of PSGL-1 and CLA expression on neutrophils treated with RPMI or deactivated bromelain.

Figure S5: Flow cytometric analysis of CLA levels on human neutrophils following bromelain treatment.

Figure S6: SDS-PAGE analysis and silver staining of untreated PSGL-1 and PSGL-1 treated with bromelain.

For **P-selectin**:
Site density = 2.96(F.I.)-194.07, R^2 = 0.92

P-selectin				
Substrate	F.I., low-3 sec	Molecules /µm²	F.I., High-30 sec	Molecules /µm²
P1	679.00	295.36	2978.00	1073.10
P2	475.00	226.34	1316.00	510.85
P3	506.00	236.83	1187.00	467.21
P4	434.00	212.47	1164.00	459.43
P5	268.00	156.32	1131.00	448.26
Average	472.40	225.46	1555.20	591.77
stdev	147.54	49.91	798.46	270.11
SEM	65.98227	22.32	357.08	120.80

Approximations:
Low P-selectin: 250 molecules/µm^2
High P-selectin: 600 molecules/µm^2

For **E-selectin**:
Site density = 5.97(F.I.)-1597.20, R^2 = 0.91

E-selectin				
slide	F.I., Low-6 sec	Molecules /µm²	F.I., High-60 sec	Molecules /µm²
E1	618.70	371.27	1088.00	449.90
E2	812.00	403.66	6805.00	1407.78
E3	541.00	358.25	801.00	401.82
Average	657.23	377.73	2898.00	753.17
stdev	139.55	23.38	2765.15	567.42
SEM	62.40811	13.50	1236.61	327.60

Approximations:
Low E-selectin: 400 molecules/µm^2
High E-selectin: 750 molecules/µm^2

Figure S1: Average fluorescence intensity and site density determination for P-selectin and E-selectin substrates. Substrates were incubated with fluorescently labeled antibodies, and fluorescence intensity units were converted to site density (molecules/µm^2) using previous reported calibration curves (1).

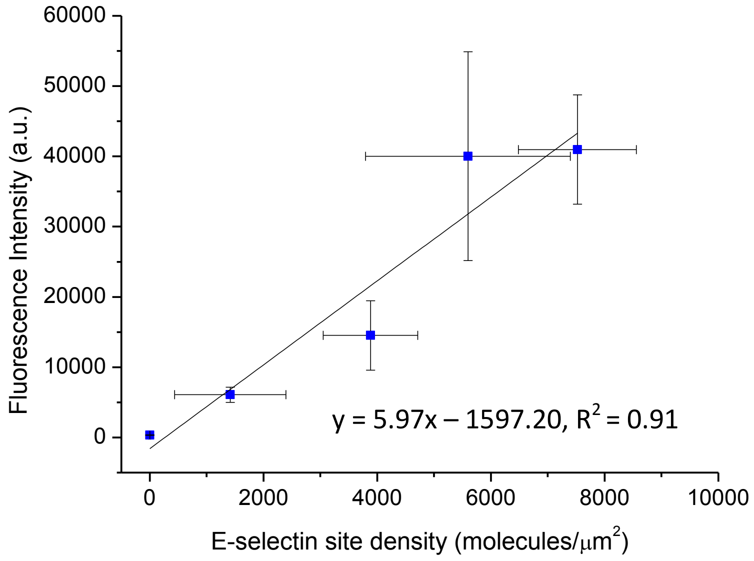

Figure S2: The relationship between fluorescence intensity and site density was determined for photoimmobilized E-selectin on BP-modified substrates. Substrates were generated in triplicate via flood UV exposures in the presence of protein solution, resulting in homogeneous substrates presenting a wide range protein site density levels. Fluorescence measurements were acquired after incubating substrates with fluorescently labeled antibodies. Briefly, we generated six replicate substrates presenting varying levels of immobilized E-selectin in a defined area on BP-modified substrates and split the substrates into two groups: one group for fluorescence analysis and the other for radioactivity analysis. For site density determination, substrates were incubated with saturating concentrations of primary monoclonal antibody (mAb) and [^{125}I]-labeled secondary polyclonal antibody (pAb). Control substrates were used to account for non-specific mAb and pAb binding. The data from both studies were correlated to determine the relationship between fluorescence intensity and site density for each protein. Using the equation from the linear regression for the resulting calibration curve, immobilized E-selectin substrates were quantified by converting data from fluorescence analyses to site density. Data is plotted as the average (± 95% C.I.) for *n*=3 substrates.

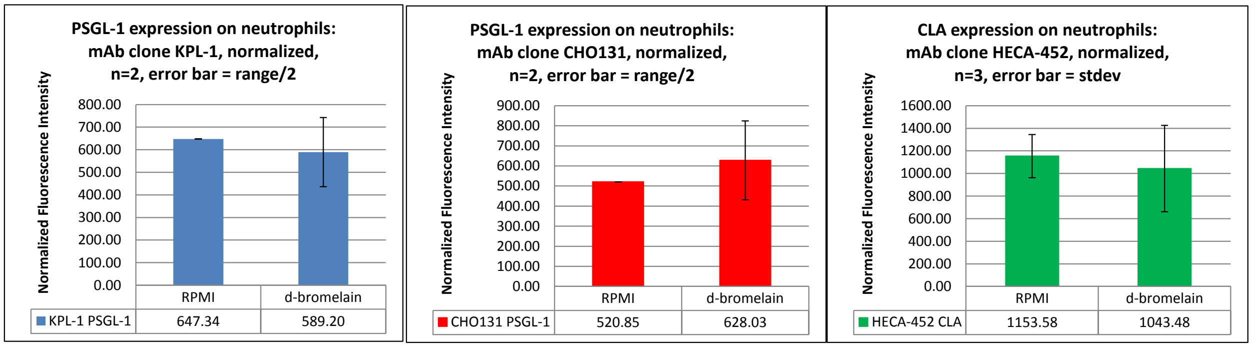

Figure S3: Flow cytometry analysis of PSGL-1 and CLA expression on neutrophils treated with RPMI or deactivated bromelain reveals that deactivated bromelain (1000 μg mL^{-1}) has no significant effect on PSGL-1 or CLA expression levels. Data presented is from 2 or 3 donors, and has been normalized to account for differences in the flow cytometry instrument parameters used in each analysis.

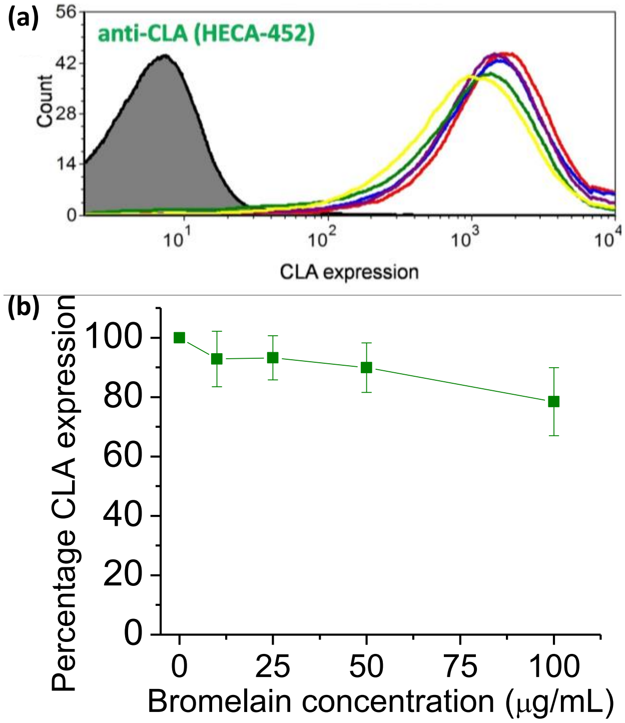

Figure S4: Flow cytometric analysis of CLA levels on human neutrophils following bromelain treatment. (a) Representative data from an analysis with anti-CLA clone HECA-452 reveals a slight decrease in PSGL-1 expression as bromelain concentration increases from 0 to 100 μg mL^{-1}. (b) A plot of the average CLA expression levels of *n*=6 donors (±SEM) following bromelain treatment ranging from 0 to 100 μg mL^{-1} reveals that bromelain causes average CLA expression to decrease by ~20%.

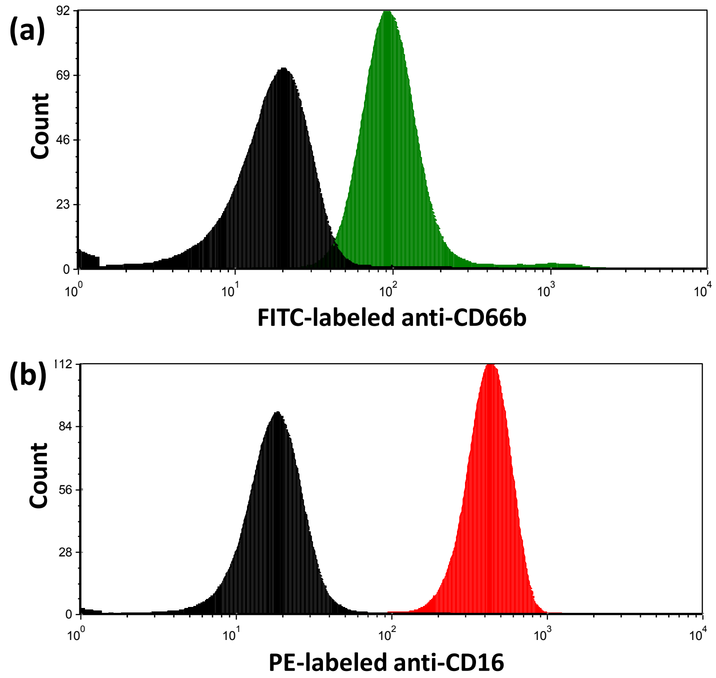

Figure S5: Flow cytometry analysis of human neutrophils for neutrophil markers (a) CD66b and (b) CD16 revealed >90% neutrophil purity in all cell samples isolated for neutrophil flow assay experiments (representative data shown). Neutrophils were isolated using traditional Ficoll-Paque density centrifugation followed by magnetic bead separation. Control sample (black) were incubated with fluorescently labeled isotype control antibodies.

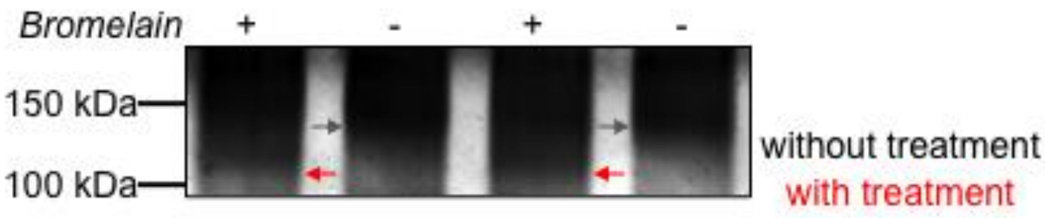

Figure S6: Bromelain proteolytically cleaves PSGL-1. SDS-PAGE analysis and silver staining of untreated PSGL-1 and PSGL-1 treated with bromelain reveals that bromelain treatment decreases the observed molecular weight.

Supplemental References

1. Herman, C.T., Potts, G.K., Michael, M.C., Tolan, N.V., and Bailey, R.C. (2011) *Integrative Biology* **3**, 779-791.
